# Mechanism of Xinfeng Capsule in the Treatment of Hypercoagulable State of Ankylosing Spondylitis Based on Data Mining and Network Pharmacology

**DOI:** 10.1155/2022/8796980

**Published:** 2022-03-17

**Authors:** Xu Li, Jian Liu, Yanyan Fang, Mingyu He, Fanfan Wang, Qi Han

**Affiliations:** ^1^Graduate School, Anhui University of Traditional Chinese Medicine, Hefei 230038, China; ^2^Department of Rheumatism Immunity, The First Affiliated Hospital, Anhui University of Chinese Medicine, Hefei 230031, China; ^3^Department of Clinical Data Center, The First Affiliated Hospital, Anhui University of Chinese Medicine, Hefei 230031, China

## Abstract

**Background:**

Ankylosing spondylitis (AS) is a rheumatism that mainly affects the axial bones and joints. Xinfeng capsule (XFC) is a preparation with a remarkable clinical effect that is used in our hospital. And it has definite curative effect and less side effects in the treatment of AS.

**Objective:**

Data mining and network pharmacology were used to analyze the efficacy of Chinese medicine Xinfeng capsule on treating the hypercoagulable state of ankylosing spondylitis and the underlying mechanism behind it.

**Methods:**

Clinical data were collected and compiled from the Department of Rheumatology and Immunology of the First Affiliated Hospital of Anhui University of Chinese Medicine. Cluster analysis was used to investigate herbs that frequently used to treat AS, Apriori module was used to analyze the association rules between herbs and laboratory indexes, and the random walk model was used to reveal the therapeutic efficacy of XFC against AS. The TCMSP database was used to acquire the active components and targets of XFC, and the GeneCards and OMIM database were used to obtain the targets of AS. Afterward, an active ingredient-target network was established and core targets were screened for; overlapping targets were screened for the protein-protein interaction (PPI) network analysis, the Gene Ontology (GO) enrichment analysis, and the Kyoto Encyclopedia of Genes and Genomes (KEGG) pathway analysis. Molecular docking was adopted to investigate the interactions between main active components and core targets.

**Results:**

Frequently used herbs could be divided into three groups, and according to the analysis of Apriori module, there is a strong correlation between XFC and the improvement of ESR and hs-CRP, and the results of the random walk model demonstrated that the effect of XFC on improving PLT, ESR, and hs-CRP was superior to the use of traditional Chinese medicine alone. In total, 103 active compounds of XFC and 59 overlapping targets were obtained. The PPI relationships were obtained through the STRING database, and 13 core targets were identified. 1786 GO enrichment results and 205 KEGG enrichment results were obtained, including NF-kappa B signaling pathway, TNF signaling pathway, and IL17 signaling pathway. The outcomes of molecular docking revealed a close relationship between the active compounds of XFC and core targets.

**Conclusion:**

This study demonstrated that XFC can effectively improve the hypercoagulable state and the inflammatory indices of AS patients through data mining, and it has a strong correlation with the clinical improvement of inflammation. The active compounds of formononetin, triptolide, quercetin, and kaempferol may be the key active components of XFC in regulating AS, possibly through inhibiting the activation of NF-kappa B signaling pathway to improve hypercoagulable state.

## 1. Introduction

Ankylosing spondylitis (AS) is an autoimmune disease that has a high incidence, mainly manifested as spinal inflammation and chronic connective tissue lesions [1]. It is closely related to the human leukocyte antigen-B27 gene (HLA-B27) [2], the incidence rate of AS is about 0.3% and is greater in males [3], and the ratio of male to female ranges from 2 : 1 to 9 : 1 [4]. The commonly used drugs are nonsteroidal anti-inflammatory drugs and sulfonamides, which are anti-inflammatory and analgesic, controlling the development of the disease and reducing the disability rate; yet there are certain side effects. There is an early understanding of AS from traditional Chinese medicine with a rich theoretical basis and clinical experience in its treatment.

Xinfeng capsule (XFC) is a preparation with a remarkable clinical effect that is used in our hospital. It is mainly composed of four herbs: Astragalus membranaceus (Huang Qi:HQ), Coix seed (Yi Yi Ren:YYR), Tripterygium wilfordii (Lei Gong Teng:LGT), and centipede (Wu Gong:WG). XFC has been used in clinic for more than 10 years, and it has definite curative effect and less side effects in the treatment of AS [5].

As a new scientific method, network pharmacology is based on the analysis of network data and system biology. The combination of traditional Chinese medicine and network pharmacology has bridged the gap between modern medicine and traditional medicine [6].

The clinical efficacy of traditional Chinese medicine on treating AS is valid, as it improves the immune inflammations indices of AS patients [7, 8], but the prescription remained complex and variable [9], which hindered the further exploration of its exact mechanism. Therefore, this study conducted data mining research on the prescriptions of traditional Chinese medicine and laboratory indicators of hospitalized patients and is aimed at exploring the mechanism of XFC used to treat ankylosing spondylitis through the method of network pharmacology, so as to provide a basis for the clinical application of XFC.

## 2. Materials and Methods

### 2.1. Materials

Clinical data from hospitalized AS patients in the Department of Rheumatology and Immunology of the First Affiliated Hospital of Anhui University of Chinese Medicine between July 2009 and June 2021 were collected and compiled. The dataset includes usage records of XFC and Chinese herbal medicines, and the value of laboratory indicators includes PLT, hs-CRP, and ESR. 1040 datasets were acquired, Among them, 710 received Chinese medicine alone as control groups, and 330 were treated with XFC combined with Chinese medicine as experimental groups.

### 2.2. Cluster Analysis

Any Chinese herbal medicine that was prescribed to individual patients was assigned 1, and those not used were assigned 0. The compatibility of Chinese herbal medicine was studied by systematic clustering in the SPSS v. 23.0 software. In cluster analysis, each herb was considered as a cluster and then combined based on their similarity to form a new class. The formula is as follows:

(1) $$\begin{array}{c} d\left(x,y\right)=\sqrt{\overset{n}{\underset{k=1}{\sum }}{\left(x_{k}-y_{k}\right)}^{2}.} \end{array}$$

### 2.3. Association Rules

Any Chinese herbal medicine that was prescribed to individual patients was assigned 1, and those not used were assigned 0; any improvement in the laboratory indices was also assigned 1, otherwise 0. The Apriori module in SPSS Clementine client v. 11.1 was used to determine the correlation between Chinese herbal medicine and laboratory indicators. The minimum support rate was 30%, the confidence level was 50%, and the lift was >1. Apriori was used to establish relationships between items. Individual drug and index were used as variables. The formula is as follows:

(2) $$\begin{array}{c} \text{Support}\left(X⟶Y\right)=\sigma \frac{\left(X\cup Y\right)}{N},\\ \text{Confidence}\left(X⟶Y\right)=\sigma \frac{\left(X\cup Y\right)}{\sigma \left(X\right)},\\ \text{Lift}\left(X⟶Y\right)=\text{confidence}\frac{\left(X\cup Y\right)}{\sigma \left(Y\right)}, \end{array}$$

where *X*⟶*Y* represents an association rule, *X* (left-hand side (LHS)) represents the set of laboratory indices, and *Y* (right-hand side (RHS)) represents herbs, and *σ*(*X*) is the probability of the itemset *X* occurring; *X* ∪ *Y* is the union of itemsets *X* and *Y*, and *σ*(*X* ∪ *Y*) is the probability when *X* and *Y* occur together. Support(*X*⟶*Y*) represents the probability of itemset (*X*, *Y*) occurring within the total itemset, and confidence(*X*⟶*Y*) is the probability of *Y* occurring in the presence of *X*. Lift is the ratio of the probability that itemset *Y* appears in the presence of *X* to the probability of *Y* appearing. Support and confidence are usually used to eliminate insignificant associations, and lift indicates the effectiveness of the association rules.

### 2.4. Random Walk Model

The random walk model evaluation in the index laboratory was realized using the Oracle 10 g tool; Peng's random walk model was used to refer [10], and according to the ideas of this model, the changing process of efficacy indicators such as symptom signs, physicochemical indices, and certain scale indicators, clinically can reflect the effects of the corresponding treatment options; hence, PLT, hs-CRP, and ESR were used as the indices for the random walk model evaluation.

### 2.5. Screening of Active Ingredients and Targets

The compounds in XFC consisting of four Chinese herbs were acquired from the Traditional Chinese Medicine Systems Pharmacology Database and Analysis Platform (TCMSP, https://tcmspw.com), which is a Chinese herbal medicine platform [11]. Based on the rules of optimal toxicokinetic ADME, drug-likeness (DL) ≥ 0.18, and oral bioavailability (OB) ≥ 30%, the bioactive components of XFC were chosen. However, the active components of centipede (WG) were zero according to the criteria; therefore, the literature was searched to supplement the compounds. Then, the targets were corrected by the UniProt database to establish the gene symbol of active compounds.

### 2.6. Acquisition of AS Associated Targets

The keyword “ankylosing spondylitis” was searched for in the GeneCards (http://www.genecards.org/) and OMIM databases (http://www.omim.org/); AS related targets were recognized and filtered, with duplicate targets deleted to establish the targets of AS. Targets of AS and XFC were collected and visualized through R 4.1.1.

### 2.7. Construction of Active Component-Target Network

Based on the aforementioned active ingredients of traditional Chinese medicine, potential targets were intersected with those related to AS, and 59 targets were obtained and introduced into the Cytoscape 3.8.2 software for constructing the active component-target network.

### 2.8. PPI Network Structure and Screening of Core Targets

Overlapping targets relating to both AS and XFC were screened to clarify the interaction between XFC and AS by R 4.1.1. Potential targets were then uploaded to the STRING database (https://string-db.org/), in which the species was set as “Homo sapiens” for retrieving a PPI network with strong confidence. The network was then analyzed in the Cytoscape 3.8.2 software, in which the CytoNCA plug-in [12] was used to identify core targets, and the “network analyzer” tool was adopted to calculate the “betweenness centrality (BC),” “degree centrality (DC),” “closeness centrality (CC),” “eigenvector centrality (EC),” “method based on local average connectivity (LAC),” and “network centrality (NC)”; then, core targets were selected through double extraction, whose criteria are as follows: BC ≥ median (BC), DC ≥ median (DC), CC ≥ median (CC), EC ≥ median (EC), LAC ≥ median (LAC), and NC ≥ median (NC).

### 2.9. Bioinformatics Analysis

Both Gene Ontology (GO) enrichment and Kyoto Encyclopedia of Genes and Genomes (KEGG) pathway analyses were carried out using the R 4.1.1 clusterProfile V3.12.0. The Go enrichment analyzed biological processes, cellular composition, and molecular functions, whereas the KEGG pathway analyzed important biological pathways.

### 2.10. Molecular Docking

The core targets and main components were selected for molecular docking. The mol2 format file of active compound structure was downloaded on the TCMSP platform, along with the 3D structure of the corresponding target from PBD (https://www.rcsb.org/), the molecular docking of the target and active components was carried out by the AutoDock 4.2.6 software, and the visualization was achieved by PyMOL 2.5.2. The best affinity and minimum binding energies are conformations as final docking results.

## 3. Results

### 3.1. Cluster Analysis of Herbs in AS Therapy

**Figure 1:**
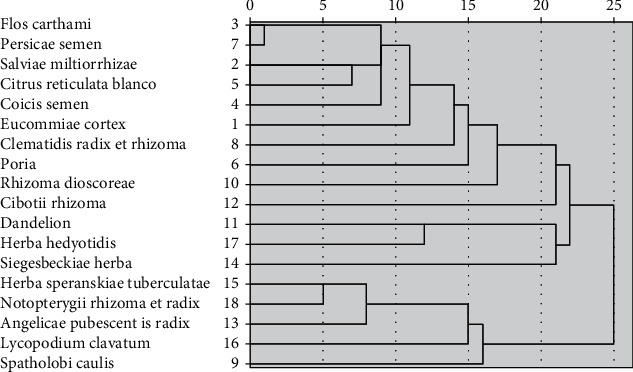
Cluster analysis of herbs used in AS.

A cluster analysis of the herbs used for therapy was conducted, at 20 Euclidean distances; herbs could be categorized into three groups (Figure 1).

### 3.2. Association Rule Analysis of Herbs and Laboratory Indices

The minimum support rate was 30%, the confidence was 50%, and the lift was >1. Apriori was used to establish relationships between herbs and laboratory indices (Table 1).

### 3.3. Random Walking Model of Laboratory Indices

**Figure 2:**
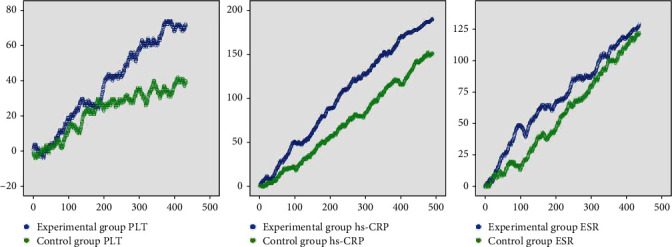
Random walking model of laboratory indices. Note: Blue line signifies the experimental group. Green line symbolizes the control group. The length of the horizontal line grows with the number of steps. Vertical line height grows with the effect of the intervention.

The PLT improvement coefficients in the control and experimental groups were 0.081 and 0.164, respectively, and the clinical significance lies within the improvement of each index, which requires 23.030 steps and 10.730 steps to be taken, respectively. The improvement coefficients of hs-CRP were 0.260 and 0.387, respectively, and the clinical significance was the need to take 6.630 and 4.330 steps, respectively, for an improvement to be seen in each of the combined indices. The improvement coefficients of ESR were 0.243 and 0.295, respectively, and the clinical significance was walking for 7.400 and 5.940 steps, respectively, for improvement in each composite measure to occur (Table 2 and Figure 2).

### 3.4. Active Components of Screening for XFC

**Figure 3:**
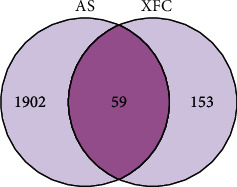
AS-XFC common targets.

The components of Astragalus membranaceus (Huang Qi:HQ), Coix seed (Yi Yi Ren:YYR), Tripterygium wilfordii (Lei Gong Teng:LGT), and centipede (Wu Gong:WG) were acquired from the TCMSP database and searched from literature. 103 active compounds and 212 potential targets of XFC were obtained. 1961 AS targets were obtained by screening the GeneCards database and OMIM database. Through inspecting the intersection of drug and disease targets, 59 overlapping targets were identified (Figure 3).

### 3.5. Active Component Target Network Construction

**Figure 4:**
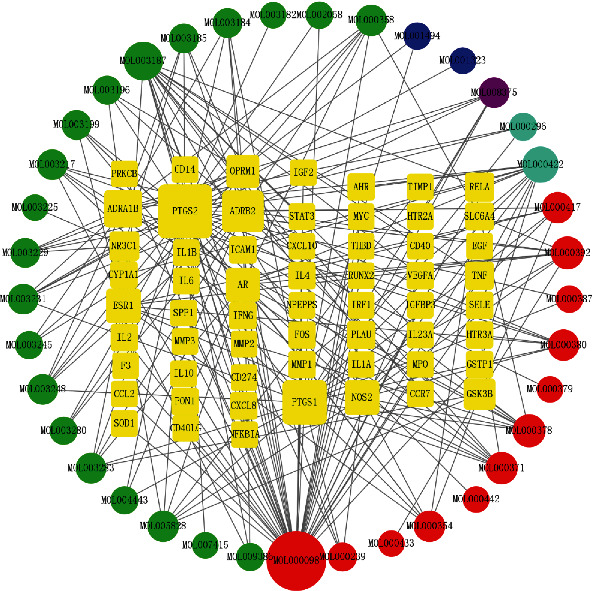
Network construction.

The active component target network, which can intuitively reflect the interactions between active ingredients and targets, was designed using the Cytoscape v3.8.2 software (Figure 4). Red nodes indicate active components of Astragalus membranaceus, blue nodes represent active components of Coix seed, green nodes symbolize active components of Tripterygium wilfordii, purple nodes symbolize active components of centipede, and light blue represents the common components between Astragalus membranaceus and Tripterygium wilfordii. The presence of an interconnection indicates a relationship between components and targets. The size of nodes indicates the magnitude of the degree value, and the greater the degree, the greater the number of nodes it is connected to, hence the greater the regulatory function of the whole network. The top four active ingredients identified from the degree value were as follows: formononetin, triptolide, quercetin, and kaempferol.

### 3.6. PPI Network Structure and Screening of Core Targets

**Figure 5:**
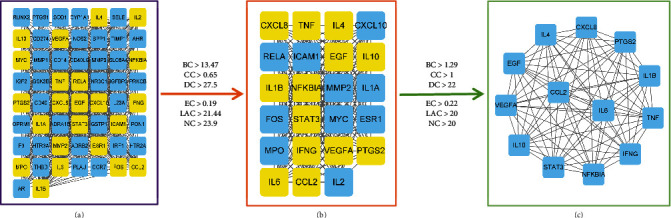
Identification of core targets.

59 overlapping targets were uploaded to the STRING database to export the TSV text. The PPI network built by Cytoscape 3.8.2 is shown in Figure 5(a), with 59 nodes and 762 edges in total. After double network topology analysis, 13 key targets that were screened out are shown in Figure 5(c): IL4, IL6, TNF, IL1*β*, VEGFA, IL10, CCL2, PTGS2, CXCL8, EGF, STAT3, NF*κ*BIA, and IFNG.

### 3.7. GO and KEGG Analyses

**Figure 6:**
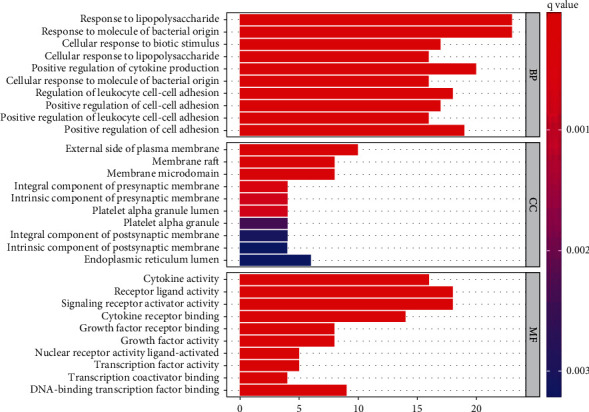
Barplots of GO analysis.

The Go analysis contained the following aspects: biological process (BP), cellular components (CC), and molecular function (MF). In the end, 1786 GO enrichment results were obtained. The upper 10 of BP, CC, and MF are shown in Figure 6. BP mainly involved responses to lipopolysaccharide, bacterially derived molecules, and cellular responses to biotic stimulus. CC was mainly related to the outer side of the plasma membrane, the membrane raft, and membrane microregion. MF was mainly involved in cytokine activity and receptor ligand activity, along with that of signaling receptor activator activity.

**Figure 7:**
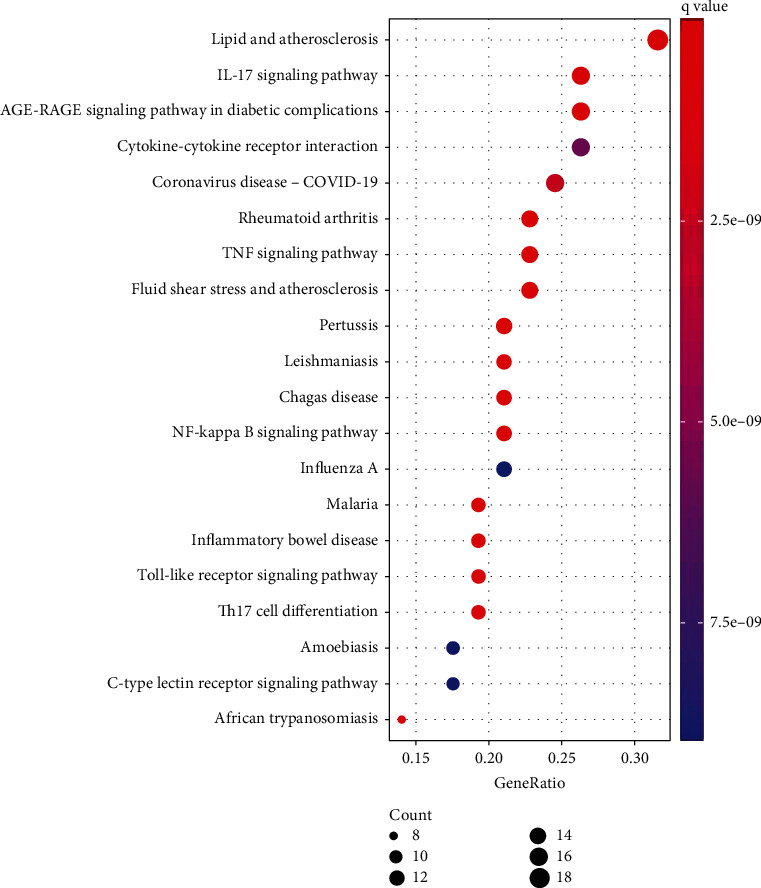
Bubble plots of KEGG analysis.

205 KEGG enrichment results were obtained, and Figure 7 demonstrates 20 important pathways (*q* value < 0.05), and some played essential roles, such as the NF-kappa B signaling pathway, TNF signaling pathway, and IL17 signaling pathways. The potential pathways were mainly involved with inflammatory responses, antiviral functions, and immune regulation.

### 3.8. Molecular Docking

**Figure 8:**
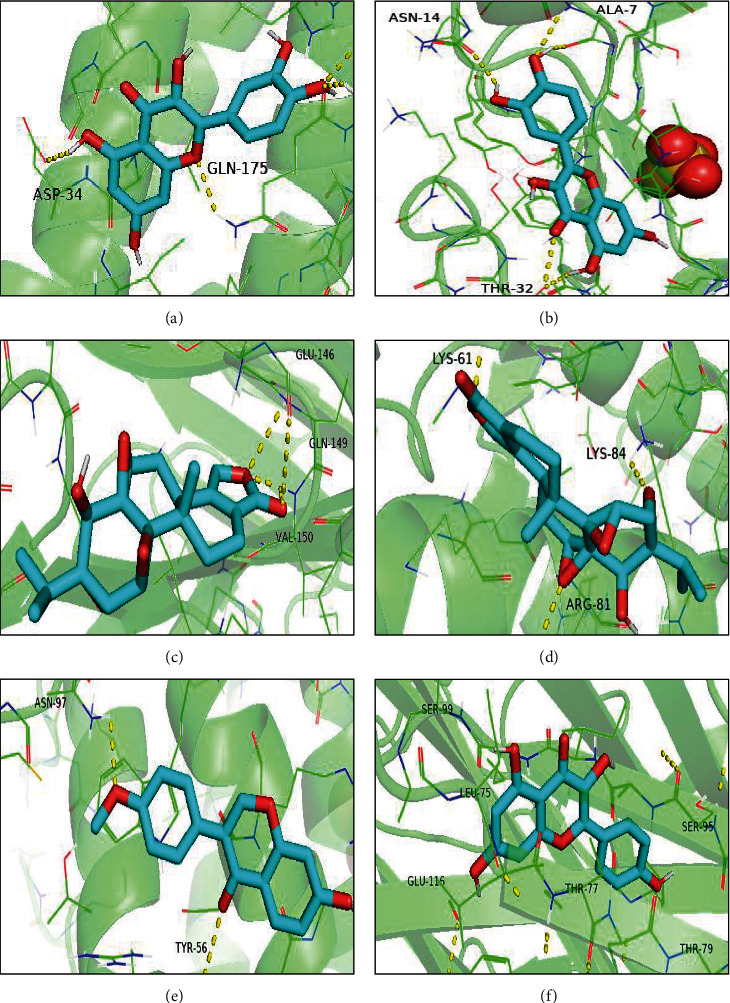
Molecular docking: (a) IL6 with quercetin, (b) CCL2 with quercetin, (c) TNF with triptolide, (d) IL4 with triptolide, (e) IL4 with formononetin, and (f) TNF with kaempferol.

The main active components, such as formononetin, triptolide, quercetin, and kaempferol, were docked with IL6, CCL2, TNF, and IL4 of the core targets. The results showed that all targets and molecules were successfully docked, as shown in Table 3. The smaller the binding energy value, the more stable the binding was. Some docking results were selected in this study, as displayed in Figure 8.

## 4. Discussion

Previous studies of our team have discovered that the formation of AS might be related to cytokine disorder, and the NF-*κ*B signaling pathway was related to overactivation [13], the clinical efficacy of traditional Chinese medicine in the treatment of AS has been demonstrated, and its use has been widely accepted due to its simple combination, flexibility, and proven positive outcome [14]; the use of traditional Chinese medicine under the guidance of the principle of syndrome differentiation and treatment yielded satisfactory curative effects for various disease manifestations during the progression of AS, such as limited lumbar and spinal activity and morning stiffness [15, 16].

The present study made use of data mining to explore the usage rules of Chinese herbal medicine for AS treatment in our hospital and verified its efficacy. Three groups of commonly used Chinese herbal combinations for AS treatment were obtained through cluster analysis, and treatment modalities mostly adopted the methods of tonifying Qi and strengthening of the spleen, dispelling wind, and dehumidification, promoting blood circulation and removing blood stasis. The results of association rules showed that XFC has a strong correlation with the improvement of ESR and hs-CRP. The improvement of PLT, ESR, and hs-CRP in the experimental group was superior to those in the control group as shown in the random walk model, and the experimental group had a lower number of walking steps compared to the control group, hence supporting the effectiveness of XFC in treating AS. Data mining results showed that XFC can effectively improve the hypercoagulable state and inflammatory indexes of as patients, regulate the hypercoagulable state and inflammatory response, and has a strong correlation with the improvement of hypercoagulable state and inflammation.

Network pharmacology [17, 18], which involves system biology and computer technology analysis, could be used to investigate the mechanism of action of drugs. In the active component-target network of XFC, 103 active compounds were selected. Four of the major compounds, including formononetin, triptolide, quercetin, and kaempferol, were recognized as active components of XFC. Triptolide can significantly reduce the severity of collagen induced arthritis. It not only has anti-inflammatory effect, but also has the ability to prevent bone destruction [19]. Formononetin is one of the major active components of Astragalus membranaceus [20], and it shows a wide range of physiological effects beneficial to health through estrogen dependent and independent mechanisms [21]. Quercetin is a natural flavonoid compound with a variety of biological activities, and it can reduce chondrocyte apoptosis and extracellular matrix degradation [22]. Kaempferol, also known as polyphenols, is a type of flavonoids seen in many plants. Studies have identified it can obviously promote the osteogenic differentiation of mesenchymal stem cells and osteoblasts [23].

The topological parameters of identifying key nodes were calculated through the PPI network, and 13 targets were found to be the core targets for XFC in the treatment of AS, including IL4, IL6, TNF, IL1*β*, VEGFA, IL10, CCL2, PTGS2, CXCL8, EGF, STAT3, NF*κ*BIA, and IFNG. These targets are related to host immunity, oxidative stress, and other pathogenic microorganisms. IL6 is a pleiotropic cytokine with numerous biological functions, which is involved in the pathogenesis of a variety of rheumatic diseases [24]. IL10 is an anti-inflammatory factor, and it has a high expression level in patients with AS, which represents an inhibitory feedback pathway and an inhibitory response to inflammation [25]. Through animal experiments, it was found that IL10 also inhibited synovial proliferation and promoted articular cartilage repair [26]. The high expression of PTGS2 can cause the proliferation of fibroblast-like synovial cells, affect the expression levels of TNF-*α*, IL1*β*, and IL6 in the serum, and aggravate the inflammatory response [27].

Go and KEGG enrichment analyses showed that XFC could control immune pathway processes. XFC was involved in biological process such as responses to lipopolysaccharide, bacterially derived molecules, cellular responses to biotic stimulus, cytokine activity, receptor ligand activity, and signaling receptor activator activity. Results of the KEGG pathway enrichment analysis illustrated that most targets were enriched in NF-kappa B signaling pathway, TNF signaling pathway, and the IL17 signaling pathway.

Previous studies from our group found that XFC could significantly improve the status of AS disease activity and immune-inflammatory indicators, and the mechanism could be contributed to inhibiting the activation of the NF-*κ*B signaling pathway [28–30]. NF-kappa B pathway has been proved to be one of the main transcription factors involved in the abnormal expression of inflammatory cytokines in AS, expressed as the abnormal activation of NF-kappa B signaling pathway and increased secretion of inflammatory cytokines lead to deposition of immune complexes and induce immune inflammatory response; abnormally active inflammation stimulates spine and sacroiliac joint, causing low back pain and morning stiffness [30].

Molecular docking was also performed to forecast complex interactions between four active components and protein targets. The binding energy further confirmed the reliability of the docking results and showed great bonding characteristics. The results demonstrated that formononetin, triptolide, quercetin, and kaempferol could have crucial roles to play in the treatment of AS.

## 5. Conclusion

The study has demonstrated that XFC can improve the hypercoagulable state and the inflammatory indices of AS patients, and it has a strong correlation with the clinical improvement of inflammation. The mechanism of XFC used to treat hypercoagulable state of AS was analyzed through network pharmacology, and its possible molecular mechanism involves multiple components. The active compounds of formononetin, triptolide, quercetin, and kaempferol may be the key components of XFC in regulating hypercoagulable state of AS and may do so through the NF-kappa B signaling pathways. However, there are some limitations in this present study, and clinical experimental validation of superior quality is needed to support the results concluded.

## Figures and Tables

**Table 1:** Association rule of herbs and laboratory indices.

Indices(LHS, *X*)	Herbs(RHS, *Y*)	Support(*X*)(%)	Confidence(*X*⟶*Y*)(%)	Lift
PLT↓	Angelicae Sinensis Radix	32.53	52.07	1.16
hs-CRP↓	XFC	31.761	66.97	1.098
hs-CRP↓	Herba Hedyotidis	40.038	66.346	1.087
ESR↓	XFC	31.761	52.424	1.039
ESR↓	Herba Hedyotidis	40.038	52.404	1.039
ESR↓	Dandelion	61.983	52.329	1.038

**Table 2:** Random walking model of laboratory indices.

Index	Group	Maximum random fluctuation	Walking positive growth rate	Random fluctuation power law value	Improvement coefficient	Comprehensive evaluation records	Ratio
PLT	Control group	66	0.044	0.327 ± 0.14	0.081	811	23.030
	Experimental group	71	0.093	0.308 ± 0.098	0.164	432	10.730
hs-CRP	Control group	254	0.151	0.417 ± 0.117	0.260	976	6.630
	Experimental group	189	0.231	0.350 ± 0.110	0.387	488	4.330
ESR	Control group	216	0.135	0.365 ± 0.118	0.243	889	7.400
	Experimental group	129	0.168	0.353 ± 0.122	0.295	436	5.940

**Table 3:** Screening docking results.

Core targets (PDB ID)	Active ingredients	Binding energy (kcal/mol)
IL6 (1ALU)	Quercetin	-7.0
CCL2 (1DOK)	Quercetin	-7.5
TNF (2ZPX)	Triptolide	-8.5
IL4 (1HZI)	Triptolide	-6.9
IL4 (1HZI)	Formononetin	-6.3
TNF (2ZPX)	Kaempferol	-8.5

## Data Availability

All the datasets are available by conduct with the corresponding author.
